# Thrombocytopenia Is an Independent Risk Factor for the Prognosis of Thrombotic Microangiopathy in Chinese Patients With Systemic Lupus Erythematosus

**DOI:** 10.3389/fmed.2021.772607

**Published:** 2021-11-08

**Authors:** Fan Yang, Junwei Tian, Linyi Peng, Li Zhang, Jia Liu, Xinping Tian, Wen Zhang, Mengtao Li, Yan Zhao, Fengchun Zhang, Xiaofeng Zeng, Li Wang, Xiaomei Leng

**Affiliations:** ^1^Department of Rheumatology and Clinical Immunology, Chinese Academy of Medical Sciences and Peking Union Medical College, National Clinical Research Center for Dermatologic and Immunologic Diseases, Ministry of Science and Technology, State Key Laboratory of Complex Severe and Rare Diseases, Peking Union Medical College Hospital, Key Laboratory of Rheumatology and Clinical Immunology, Ministry of Education, Beijing, China; ^2^Department of Rheumatology and Nephrology, The First Hospital of Changsha, Hunan, China; ^3^Department of Medical Science and Technology Evaluation, Institute of Medical Information, Chinese Academy of Medical Sciences and Peking Union Medical College, Beijing, China

**Keywords:** systemic lupus erythematosus, thrombotic microangiopathy, clinical characteristics, thrombocytopenia, risk factor

## Abstract

**Objectives:** This study aims to describe clinical characteristics and outcome of thrombotic microangiopathy (TMA) in Chinese patients with systemic lupus erythematosus (SLE), and investigate the risk factors.

**Methods:** We conducted a retrospective single-center cohort and enrolled patients of TMA associated with SLE between January 2015 and December 2018. Demographic characteristics, clinical features, laboratory profiles, therapeutic strategies, and outcomes were collected. The risk factors of TMA in patients with SLE for mortality using multivariate analysis were estimated.

**Results:** A total of 119 patients with a diagnosis of TMA were enrolled within the study period in our center, and SLE was found in 72 (60.5%) patients. The mean age was 29.2 ± 10.1 and 65 (92.3%) were women. Only 15 patients were found with definite causes, the other 57 cases remained with unclear reasons. Sixty-two patients got improved, while 10 patients died after treatment (mortality rate: 13.9%). Compared with the survival group, the deceased group had a higher prevalence of neuropsychiatric manifestations, infection with two or more sites, increased levels of C-reaction protein (CRP) and D-Dimer, and decreased platelet count. Multivariate analysis showed that the decrease of platelet count is the independent risk factor for in-hospital mortality for TMA in patients with SLE. The receiver operating characteristic (ROC) curve analysis displayed that a cutoff value of <18 × 10^9^/L for platelet count could significantly increase the risk of death.

**Conclusions:** Thrombotic microangiopathy often occurs in patients with active SLE with high mortality (13.9%), and thrombocytopenia, especially when the platelet count is lower than 18 × 10^9^/L, is the risk factor for death.

## Introduction

Thrombotic microangiopathy (TMA) is an uncommon, life-threatening disease that involves multiple systems by microvascular occlusion and manifests as microangiopathic hemolytic anemia (MAHA), thrombocytopenia, and organ injury. Several systemic diseases have been associated with TMA, of which thrombocytopenic purpura (TTP) and hemolytic uremic syndrome (HUS) are the most commonly described. Autoimmune diseases such as systemic lupus erythematosus (SLE), scleroderma renal crisis, anti-phospholipid syndrome (APS), or malignant hypertension can also cause TMA (1). Although these diseases are remarkably diverse, they are united by common, defining clinical and pathological features.

Systemic lupus erythematosus is an autoimmune disease characterized by the production of autoantibodies. Previous researches regarding TMA developing in patients with SLE have been few and the diagnosis of TMA in patients with SLE might be challenging, possibly due to the insufficient awareness of clinicians and overlapping clinical features including hemolytic anemia, thrombocytopenia, neurological deficits, and renal involvement of the two diseases. Delayed diagnosis might cause fatal results (2). In recent years, more attention has been paid to these diseases, but the systematic and deeper investigation lacked, and data from large studies on patients with SLE with TMA were limited. Further investigation about how SLE intersects with the spectrum of TMA as well as early identification, treatment, and prognosis may allow to be discussed.

In this study, we investigated to determine the clinical characteristics of TMA in patients with SLE. Besides, we attempted to identify the clinical outcome and risk factors for mortality of TMA associated with patients with SLE.

## Patients and Methods

### Patients

We retrospectively reviewed the data of inpatients diagnosed as having TMA and SLE at Peking Union Medical College Hospital between January 2015 and December 2018. All patients met the 1997 American College of Rheumatology (ACR) revised criteria for SLE (3) or the 2012 SLE International Collaborating Clinics (SLICC) classification criteria, and confirmed by at least two rheumatologists (4). Thrombotic microangiopathy was diagnosed by the following criteria: (1) presence of MAHA, in accompany with an increase of lactate dehydrogenase (LDH) value and fragmentation of erythrocytes on peripheral blood smear; (2) thrombocytopenia in the absence of other known causes; (3) with or without organ damage (5). Patients with kidney biopsies showing TMA and confirmed by at least two nephrologists were enrolled too (6). Renal TMA was defined as an endothelial cell swelling, lumen narrowing, or obliteration, and thrombi formation in interlobular artery, arteriole, and glomerular capillary lesions (7). The study was undertaken in accordance with the Declaration of Helsinki and approved by the Ethics Committee of Peking Union Medical College Hospital in Beijing, China (S-K1631).

### Data Collection

Demographic characteristics including age and sex, SLE disease activities, various clinical features, laboratory profiles, therapeutic strategies, and outcomes were collected from the medical records and reviewed. The systemic lupus erythematosus disease activity index (SLEDAI) was used to assess disease activity of SLE, and the following activity categories have been defined based on SLEDAI scores: no activity (SLEDAI 0–4), mild activity (SLEDAI 5–9), moderate activity (SLEDAI 10–14), and high activity (SLEDAI ≥15) (8). Autoantibodies associated with SLE were also evaluated, including anti-nuclear antibodies (ANA), anti-SSA/SSB antibodies, anti-dsDNA antibodies, anti-Sm antibodies, anti-ribonucleoprotein (RNP) antibodies, and anti-phospholipid antibodies (anti-cardiolipin antibodies, anti-β2 glycoprotein-I antibodies, and lupus anti-coagulant). ADAMTS13 (a disintegrin and metalloproteinase with a thrombospondin type 1 motif, member 13) activity and renal histopathology were also collected when available.

### Statistical Analysis

Quantitative variables were reported as the mean ± SD, and median with range (minimum, maximum). Comparisons of descriptive data between groups were performed by Student's *t*-test or Mann-Whitney U-test. Categorical data were compared using the chi-square or Fisher exact test. *P* < 0.05 was considered to indicate a statistically significant difference. Variables were entered into the univariable (UV) logistic regression model. A multivariate logistic regression model was then constructed using a stepwise forward selection procedure among those candidate variables with the significance level *p* < 0.10 in the UV logistic regression analysis. Odds ratios (ORs) and 95% CIs were calculated. The receiver operating characteristic (ROC) curve analysis was used to evaluate the cutoff point of laboratory data to predict the risk of death. Statistical analysis system(SAS) statistical software was used for all analyses.

## Results

### Demographic, Clinical, and Laboratory Profiles

**Figure 1:**
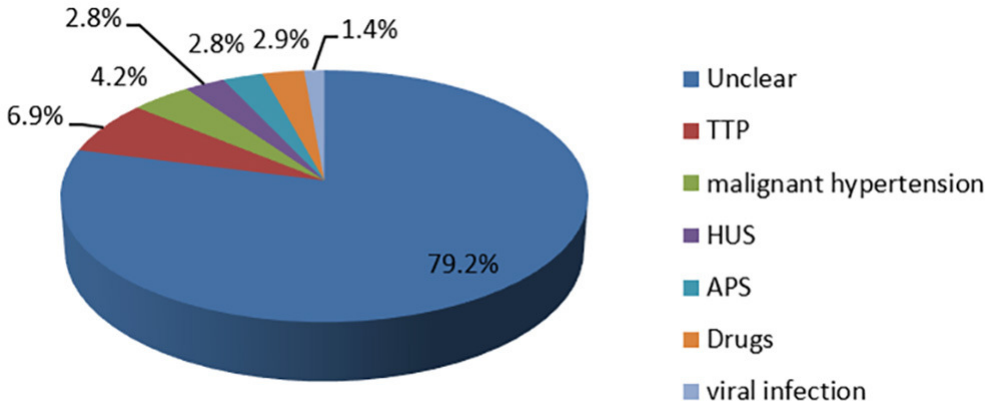
The reasons for TMA in 72 patients. Fifteen patients (20.8%) were found with definite causes, the other 57 cases (79.2%) remained with unclear reasons.

We retrieved a total of 119 inpatients with a diagnosis of TMA enrolled within the study period in our center, and SLE was found in 72 (60.5%) patients. The mean age was 29.2 ± 10.1 and 65 (92.3%) were females. The mean SLEDAI score was 18.1 ± 6.4, and patients with mild, moderate, and high disease activity accounted for 9.7, 19.4, and 70.9%, respectively. As shown in Figure 1, only 15 patients were found with clear reasons, including five with TTP, three with malignant hypertension, two with HUS, two with APS, two with drugs, and one with a viral infection, the other 57 patients remained with unclear reasons. Besides, we also analyzed the possible causes of TMA in patients with non-SLE (Supplementary Figure 1).

The clinical manifestations included fever in 47 (65.3%) cases and rash in 31 (43.1%) cases, while arthritis/arthrodynia showed lower frequency (27, 37.5%). Renal involvement was found in all patients, of which 71 had proteinuria, 69 had renal insufficiency, and 67 had hematuria. More than 95% of patients (71/72) were with hematological involvement, of which 70 had anemia, 67 had thrombocytopenia, and 60 had schistocyte in peripheral blood smears. Hypertension was recorded in 65 cases. Forty-three of patients (59.7%) had respiratory symptoms of dyspnea (40), hemoptysis (9), and chest pain (4). Neuropsychiatric manifestations were confirmed in 32 (44.4%) cases, most of them were presented as disturbance of consciousness (including nine cases of delirium, six cases of lethargy, cases of apathy, and one case of coma), followed by epilepsy (10), headache (11), hemiplegia (1), and aphasia (1). Twenty-six (35.1%) had digestive manifestations of vomiting (12), diarrhea (9), and abdominal pain (8) (Table 1).

**Table 1:** Frequencies of clinical features in 72 patients.

**Features**	**No. (%) of patients**
Fever	47 (65.3%)
Rash	31 (43.1%)
Arthritis/arthrodynia	27 (37.5%)
Renal involvement	72 (100.0%)
Proteinuria	71 (98.6%)
Hematuria	67 (93.1%)
Hematological involvement	71 (98.6%)
Anemia	70 (97.2%)
Thrombocytopenia	67 (93.1%)
Schistocyte in peripheral blood smears	60 (83.3%)
Respiratory symptoms	43 (59.7%)
Dyspnea	40 (55.6%)
Hemoptysis	11 (15.3%)
Chest pain	4 (5.6%)
Neuropsychiatric manifestations	32 (44.4%)
Disturbance of consciousness	21 (29.2%)
Delirium	9 (12.5%)
Lethargy	6 (8.3%)
Apathy	5 (69.4%)
Coma	1 (1.4%)
Epilepsy	13 (18.1%)
Headache	12 (16.7%)
Hemiplegia	1 (13.9%)
Aphasia	1 (13.9%)
Digestive manifestations	26 (52.0%)
Vomiting	15 (20.8%)
Diarrhea	11 (15.3%)
Abdominal pain	8 (11.1%)
Hypertension	65 (90.3%)

In 70.0% of the patients (51/72), infection was detected simultaneously, patients of whom infective sources with two or more sites were found in 29 cases. The principal sites of infection were in the respiratory tract (32), next were in the urinary tract (6), others included gynecological disease, pancreas, abdomen, skin and soft tissue, oral mucosa, catheter-related bloodstream infection, and so on.

As for laboratory profiles, hemoglobin drop was observed in 70 (97.2%) cases, yielding a median value of 6 (3.5–13.1) g/L. Thrombocytopenia (<100 × 10^9^/L) was observed in 67 (93.1%) cases with a mean platelet count of 34.5 (1–188) × 10^9^/L. Creatinine elevation (>90 μmol/L) was presented in 69 out of 72 patients (96.4%) with a median value of 303 (64–1,102) μmol/L. Laboratory findings also showed increased levels of erythrocyte sedimentation rate (ESR) and C-reaction protein (CRP) (44 and 45 patients, respectively) and decreased complement (C3 in 65 cases, C4 in 49 cases). The most frequent antibody was anti-dsDNA and anti-SSA in 42 (58.3%) cases, respectively, followed by anti-Sm in 16 (22.2%) cases and anti-rRNP in 13 (18.1%) cases at the time of TMA diagnosis. Besides, the anti-phospholipid antibody was found in 13 patients (lupus anti-coagulant in 10, anti-cardiolipin antibody in 8, and anti-β2-glycoprotein antibody in 11 cases). ADAMTS activity and inhibitor were tested in 34 (47.2%) patients, five of whom exhibited positive results.

A total of 24 patients underwent renal biopsy in this study. Twenty-three patients were diagnosed with lupus nephritis (LN) based on pathological diagnosis, of which 14 patients coexisted with renal TMA. According to the 2003 classification of LN, 18 patients were classified as Class IV (78.3%, including two as Class IV+V), two patients as Class I, and Class II, III, V were found in one case, respectively. Besides, one patient with a 6-year-history of diabetes was diagnosed with diabetic nephropathy and TMA. His clinical diagnosis of SLE is definite, although without renal involvement.

### Treatments

All patients received corticosteroids and intravenous corticosteroid pulse therapy (CPT), which was defined as treatment with more than 250 mg prednisone or its equivalent per day for one or more days, was given to almost three-quarters of the patients (54/72, 75.0%). The other patients received 0.3–2 mg/kg of prednisone or equivalent per day as a starting dose. Forty-seven patients used two or more immunosuppressive agents, of which cyclophosphamide (CTX), mycophenolate mofetil (MMF), and tacrolimus were the three most frequently used, received by 87.5 (63), 38.89 (28), and 9.7% (7) of the patients, respectively. Six patients were treated with a biologic agent, with rituximab (RTX) being the selected agent. Other treatments included intravenous immunoglobulin in 41 (56.9%) and anti-coagulation in six (8.3%) patients. Plasma exchange (PE) was performed in over half of the patients (42, 58.3%), with the therapeutic number 1–21 times.

### Clinical Outcomes and Risk Factors for Mortality

After treatment, 62 patients got improved (defining as the survival group) and 10 patients died (defining as the deceased group). Comparisons of clinical profiles between these two groups are shown in Table 2. Our data displayed that compared with the survival group, the deceased group had a higher prevalence of neuropsychiatric manifestations especially disturbance of consciousness and infection in two or more sites (*P* < 0.05). Significantly higher levels of CRP and D-Dimer were also found, while the platelet count was markedly lower in the deceased group (*P* < 0.05). There were no differences in age, gender, SLEDAI scores, and other laboratory profiles including hemoglobin, serum complement level (C3 and C4), creatinine, the percentage of reticulocyte, albumin, and ESR between the two groups.

**Table 2:** Comparisons of clinical profiles between the survival and the deceased group.

**Items**	**Survived (*n* = 62)**	**Deceased (*n* = 10)**	***P*-value**
Gender (M/F)	6/56	1/9	1.000
Age (years)	28.7 ± 12.1	39.1 ± 18.8	0.095
Fever	42 (67.7%)	5 (50.0%)	0.462
Neuropsychiatric manifestations	24 (38.7%)	8 (80.0%)	0.019*
Disturbance of consciousness	9 (14.5%)	8 (80.0%)	<0.001*
Epilepsy	11 (17.7%)	2 (20.0%)	1
Infection with two or more sites	21 (33.9%)	8 (80.0%)	0.012*
Hemoglobin (g/L)	62.3 ± 15.5	55.4 ± 12.0	0.328
Reticulocyte (%)	5.5 ± 4.3	7.3 ± 6.6	0.631
Platelet count (*10^9^/L)	47.8 ± 39.2	12.5 ± 12.8	<0.001*
Creatinine (μmol/L)	363.8 ± 224.7	265.1 ± 164.4	0.196
Albumin (g/L)	29.6 ± 5.7	28.9 ± 4.3	0.712
LDH (U/L)	747.7 ± 728.7	1,047.5 ± 677.8	0.105
ESR (mm/1 h)	31.5 ± 23.5	45.3 ± 42.4	0.547
CRP (mg/L)	5.5 ± 8.4	36.2 ± 73.9	0.001*
D-Dimer (mg/L)	5.5 ± 9.0	15.67 ± 18.4	0.003*
C3 (g/L)	0.5 ± 0.2	0.4 ± 0.2	0.510
C4 (g/L)	0.1 ± 0.1	0.1 ± 0.1	0.379
SLEDAI	18.0 ± 6.4	18.9 ± 6.4	0.675

*Values in bold are statistically significant at p < 0.05. Results are shown as n (%) of patients and the Mean ± SD. LDH, lactate dehydrogenase; ESR, erythrocyte sedimentation rate; CRP, C-reaction protein; SLEDAI, systemic lupus erythematosus disease activity index*.

Multivariate analysis using a logistic regression model showed that the decrease of platelet count is the only independent risk factor for in-hospital mortality for TMA in patients with SLE (OR, 0.936; 95% CI, 0.879–0.998; *P* = 0.042), while the prevalence of neuropsychiatric manifestation, infection with two or more sites, and levels of CRP and D-Dimer are not the risk factors associated with the death of TMA in patients with SLE (Table 3).

**Table 3:** Risk factors for in-hospital mortality in patients with SLE with TMA.

**Items**	**OR (95% CI)**	***P*-value**
Neuropsychiatric manifestations	3.339 (0.514,21.699)	0.207
Infection with two or more sites	3.013 (0.403,22.525)	0.283
Platelet count (*10^9^/L)	0.936 (0.879, 0.998)	0.042*
CRP (mg/L)	1.057 (0.964, 1.158)	0.241
D-dimer (mg/L)	1.005 (0.947, 1.065)	0.878

*CRP, C-reaction protein; OR, odds ratio; CI, confidence interval. Values in bold are statistically significant at p < 0.05*.

**Figure 2:**
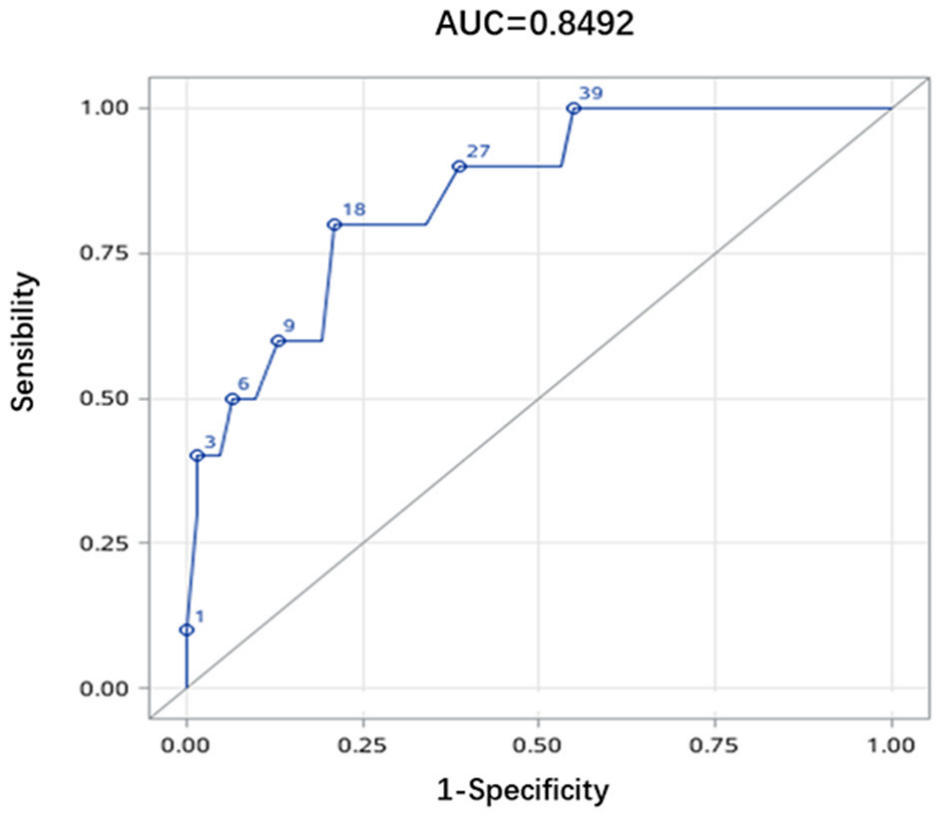
Receiver operating characteristics curve. Receiver operating characteristics (ROC) curve analysis demonstrated an area under the curve of 0.8472 for the platelet count. An optimal cutoff value of platelet count was 18 × 10^9^/L, giving the sensitivity of 80.0% and the specificity of 79.0%. The Youden Index was 0.59.

The ROC curve was calculated to determine a cutoff value of platelet count for the prediction of death, and the result is demonstrated in Figure 2. An area under the curve (AUC) >0.8 indicated good assessment accuracy. The ROC curve analysis showed that a cutoff value of <18 × 10^9^/L for platelet count may suggest a significantly increased risk of death in TMA associated with SLE.

Therapeutic modalities between the survival and deceased group were compared too. We investigated therapeutic combinations of PE and glucocorticoid or CTX to determine which had more benefits on patients. However, there were no differences between these two groups (Table 4).

**Table 4:** Comparisons of treatment between the survival and the deceased group.

	**PE**	**CPT + PE**	**CTX + PE**	**CPT + CTX + PE**
Survived (*n* = 62)	31 (50.0%)	26 (41.9%)	29 (46.8%)	29 (46.8%)
Deceased/Lost (*n* = 10)	9 (90.0%)	5 (50.0%)	4 (40.0%)	3 (30.0%)
*P*-value	0.138	0.894	0.955	0.517

*PE, plasma exchange; CPT, corticosteroid pulse therapy; CTX, cyclophosphamide*.

## Discussions

The occurrence of TMA developing in patients with SLE was rare and most publications were case reports several years ago. According to a study from Taiwan in 2011, the incidence rate of TMA in patients with SLE was only 0.1% (13). Lately, more attention has been paid to these diseases, and recent studies have reported that the prevalence of TMA in patients with SLE is up to 25.4% (14). As for the incidence of SLE in patients with TMA, Fujimura et al. reviewed 919 patients of TMA from 426 medical institutions in Japan, and 92 cases of SLE were found, accounting for 10.01% (9). Furthermore, TMA-associated SLE is a life-threatening condition with high mortality, which is as high as 90% in untreated patients (9). Even with widespread administration of PE therapy, the rate still ranges from 33.9 to 62.5% in various studies (11). In this study, we reported morbidity of SLE of 60.5% among hospitalized patients with TMA, which is higher than the above researches, which potentially reflects that SLE is one of the major causes of TMA.

The cause of TMA in SLE remains unclear and may be multifactorial, which might be attributed to APS, TTP, HUS, malignant hypertension, pregnancy, scleroderma, drugs, and so on. In our study, only 15 patients were found with clear reasons, including five with TTP, three with malignant hypertension, two with HUS, two with APS, two with drugs, and one with the viral infection. The other 57 patients only presented with clinical and pathological evidence of TMA, which was consistent with Song'study, where 29 out of 36 cases in SLE with patients with TMA only confirmed by renal biopsy and with unknown reasons (6).

Thrombotic microangiopathy often occurs in patients with active SLE (10), and active SLE disease has been considered as an independent risk factor for the development of TMA. Kwok et al. analyzed TMA in Korean patients with SLE and found that active SLE disease activity (SLEDAI >10) can be the major factor for TMA (15). This is similar to our current report, in which 90.28% of patients with TMA had moderate to high disease activity of SLE (SLEDAI >10).

The pathogenesis of TMA in SLE remains to be elucidated and may be multifactorial, including low ADAMTS13 levels, infection, drugs, etc. Mannucci et al. reported that a low serum level of ADAMTS13 was also noted in SLE. Production of autoantibodies plays a significant role in the pathogenesis of SLE and autoantibodies of ADAMTS13 are responsible for most ADAMTS13 deficiency (12). Besides, the activation of different complement pathways induced by autoantibodies also participates in the pathogenesis (6). In addition, infections can trigger TMA in patients with SLE directly or by increasing SLE disease activity. Some drugs like calcineurin inhibitor toxicity have been proved to induce TMA. Microthrombosis mediated by anti-phospholipid antibodies may also participate in the pathogenesis (16). In our study, the above-mentioned reasons have all been found, but generally, it is difficult to determine the exact cause of TMA in clinical practice because TMA is a clinical-pathological syndrome mixed with multiple reasons. Further study about pathogenesis for TMA in patients with SLE is needed to explore deeper.

Renal pathology often presents diffuse proliferative LN with subcutaneous immune complex deposition (Class IV LN) simultaneously in patients with TMA (6), and patients with Class IV LN have higher mortality and lower renal survival rate (17). In this study, LN with Class IV was found in 18 patients (78.3%), which was the main pathological type, sharing similarities with earlier series (18).

Therapeutic approaches of TMA in patients with SLE are highly variable. In addition to traditional therapies like glucocorticoids and immunosuppressants, Eculizumab, which can prevent C5b from the formation of the membrane attack complex, has shown effects in patients with TTP in SLE (19). Rituximab exposure may be an independent protective factor for the short-term survival of patients with TMA and SLE (20). Plasma exchange has been proved to improve TMA in patients with SLE and should be given when TMA features are severe or SLE disease activity is pretty high (21). Seven to fourteen courses of PE have been commonly suggested in the treatment of TMA (22). As observed in our study, in addition to the backbones of initial treatments like steroids and immunosuppressive agents, more than half of the patients received PE, but there was no significant difference between the survival and deceased group, possibly related to the shorter observation and fewer times of PE in some patients. In addition, six patients were treated with RTX, but the dosage and course were different, thus, the further assessment was unavailable.

Thrombotic microangiopathy associated with SLE is a life-threatening syndrome and the mortality in our cohort was 13.9%, lower than previous researches, but significantly higher than in-hospital mortality of 7.0% reported by Fei's study (23). Several specified organ-involved subtypes of SLE including neuropsychiatric systemic lupus erythematosus (NPSLE), hemocytopenia, and myocarditis were reported to have a worse prognosis. However, the mortalities reported in our center were lower than TMA (4.0–8.2%, 0.7, and 12.7%, respectively) (24, 25), indicating that TMA is one of the potentially fatal performances in SLE. The documents showed that age, hypocomplementemia, infection, and renal failure are associated with mortality in patients with TMA of SLE (22). According to our study, patients in the deceased group had higher rates of neurological symptoms, infections with more than two sites, increased levels of CRP and D-dimer, and lower platelet count. Multivariate analysis showed that thrombocytopenia is the independent risk factor for in-hospital mortality, especially when the platelet count is <18 × 10^9^/L, which was consistent with previous study in China. This could be explained as follows: First, multiple factors like anti-ADAMTS13 autoantibodies and vascular wall inflammation in patients with SLE, infectious triggers, and many other reasons lead to significantly decreased platelet count, which can cause excessive bleeding all over the body. Second, low platelet count potentially suggests severe underlying disease, thus, complications related to hemodialysis and organ dysfunction occurred subsequently, indicating the poor remission.

Our study has several limitations. First, it was a retrospective study and unable to control treatment options, thus, the conclusions may be of limited value. A multicenter prospective study may help to address this limitation. Second, ADAMTS 13 activity or its inhibitor was not checked in all cases because measurement of ADAMTS 13 or inhibitor was not available conveniently. Besides, only 33.3% of patients with SLE with TMA had a kidney biopsy in our study, representing that majority of TMA was diagnosed by clinical diagnosis, not a pathological diagnosis. Finally, the incidence of TMA in patients with SLE may be underestimated, as less severe cases may have been managed in the outpatient setting. It would thus follow that the associated factors and morbidity identified in this study are specific to patients with SLE requiring hospitalization.

## Conclusions

This is the largest cohort to investigate TMA in patients with SLE in China to our knowledge. We found that SLE is common pathogenesis of TMA, which usually occurred in the active stage of SLE for an uncertain reason. Besides, it is associated with significantly higher mortality. Thrombotic microangiopathy developing in patients with SLE can be fatal, especially with the decreased platelet count. When the platelet count is lower than 18 × 10^9^/L, the risk of death is significantly higher. Therefore, every rheumatologist should keep a high suspicion of TMA when managing patients with SLE with thrombocytopenia.

## Data Availability Statement

The raw data supporting the conclusions of this article will be made available by the authors, without undue reservation.

## Ethics Statement

The studies involving human participants were reviewed and approved by the Ethics Committee of Peking Union Medical College Hospital in Beijing, China. Written informed consent for participation was not required for this study in accordance with the national legislation and the institutional requirements.

## Author Contributions

FY and JT collected the clinical data and drafted the manuscript. LW and XL provided guidance and reviewed the manuscript. JL performed the data analysis. LP, LZ, XT, WZ, ML, YZ, FZ, and XZ contributed to study design and data analysis. All the authors contributed to the article and approved the submitted version.

## Funding

This study was supported by the Chinese National Key Technology R and D Program, Ministry of Science and Technology (Nos. 2017YFC0907601, 2017YFC0907602, and 2017YFC0907603); Beijing Municipal Science and Technology Commission (Nos. Z201100005520022, Z201100005520023, Z201100005520025, Z201100005520026, and Z201100005520027), CAMS Innovation Fund for Medical Sciences (CIFMS) (2019-I2M-2-008), and the National Basic Research Program of China (973 Program) (No. 2014CB541801).

## Conflict of Interest

The authors declare that the research was conducted in the absence of any commercial or financial relationships that could be construed as a potential conflict of interest.

## Publisher's Note

All claims expressed in this article are solely those of the authors and do not necessarily represent those of their affiliated organizations, or those of the publisher, the editors and the reviewers. Any product that may be evaluated in this article, or claim that may be made by its manufacturer, is not guaranteed or endorsed by the publisher.
