# Supplementary material for: Thrombocytopenia Is an Independent Risk Factor for the Prognosis of Thrombotic Microangiopathy in Chinese Patients With Systemic Lupus Erythematosus
Source: Front Med (Lausanne). 2021 Nov 8;8:772607. doi: 10.3389/fmed.2021.772607 (PMC8606658; doi:10.3389/fmed.2021.772607)
Supplement: Supplementary file 1 [file Table_1.DOCX]

Supplementary Material

## Supplementary Figure 1 The causes of TMA in non-SLE patients

**Supplementary Figure 1.** Among the 47 patients, there were several clear clinical reasons for TMA as follows: 15 patients with other autoimmune diseases except SLE(oAID, 31.9%), of which 5 patients were diagnosed as cleroderma renal crisis(SRC), 8 patients with infections(17.0%), 5 patients with pregnancy(10.6%), 4 patients with glomerulonephritis(8.6%), 2 patients with primary APS(4.3%), 2 patients with malignant hypertension(4.3%), 1 patient with malignancy(2.1%), 1 patient with inherited ADAMTS13 deficiency (2.1%), 1 patient with complement factor I deficiency(2.1%). The other 8 patients had no identified causes(17.0%).
